# The effects of resident work hours on well‐being, performance, and education: A review from a Japanese perspective

**DOI:** 10.1002/jgf2.649

**Published:** 2023-09-21

**Authors:** Kazuya Nagasaki, Hiroyuki Kobayashi

**Affiliations:** ^1^ Department of Internal Medicine, Mito Kyodo General Hospital University of Tsukuba Ibaraki Japan

**Keywords:** medical education, patient safety, postgraduate resident, resident work hours, well‐being, workstyle reform

## Abstract

This article examines the impact of working‐hour restrictions on the well‐being, performance, and education of medical residents in Japan. Despite Japan's plan to introduce new regulations for resident working hours by 2024, there is still an ongoing debate regarding their appropriateness. This review provides a comprehensive overview of the current regulations of resident working hours worldwide, with a specific focus on weekly hours. The varying regulations are highlighted, including the 80‐hour‐per‐week regulation in the United States and the 48‐h‐per‐week regulation in the European Union influencing other regions. The article also discusses the effectiveness of working‐hour restrictions on residents' mental health, with shorter working hours having potentially greater benefits. However, the impacts on medical safety and resident education are mixed, and further reduction in working hours must be carefully considered to avoid adverse effects. The planned changes to working‐hour limits for residents in Japan offer a unique opportunity to gain new evidence on the impact of such regulations, which will be of interest to policymakers and researchers worldwide.

## 1 INTRODUCTION

Medical residents are physicians in training. In Japan, all medical school graduates who wish to become clinicians, regardless of their desired specialty, must undergo 2 years of clinical training to acquire basic clinical skills.^1^, ^2^ While medical residents are still learning, they are considered an essential part of the healthcare workforce, providing vital care to patients across various clinical settings. However, their long and demanding working hours have been a subject of debate for decades. According to a survey conducted by the Ministry of Health, Labour and Welfare (MHLW) in 2019, physicians in their twenties constituted the highest percentage (13.1%) of all generations of physicians in Japan who work more than 80 h weekly.^3^ In the past two decades, a growing global consensus has emerged that resident work hours should be limited to safeguard patient safety and promote resident well‐being because of the increasing concerns about the impact of extended work hours.

Although Japan is lagging in implementing such measures, resident work‐hour restrictions are expected to be introduced in 2024. The traditional medical education system in Japan emphasizes a culture of hard work and dedication. Remarkably, residents were not legally recognized as workers until 2005, over 30 years after the commencement of the residency training system in Japan in 1968.^1^, ^4^ In 2004, the current clinical training system became mandatory, requiring physicians who complete 6 years of medical school and wish to become clinicians to undergo 2 years of training with the goal of acquiring basic skills and regardless of their desired specialty.^1^ The impetus for limiting work hours for residents in Japan stemmed from the work style reform initiated by the government in 2018.^5^ As part of this reform, the Japanese MHLW introduced new regulations limiting resident work hours to 80 h weekly, with a maximum shift length of 28 h.^6^ Although this limit is comparable to those of other countries, it is substantially longer than the maximum working hours for all workers and general physicians. Moreover, the permissible overtime level exceeds the threshold for “karoshi”, which is the point at which compensation for death because of overwork is granted.^7^ As a result, there is an ongoing debate in Japan regarding the appropriateness of the maximum work hours for residents.

The impact of work‐hour restrictions is complex and depends on various factors. A large body of literature evaluates the impact of resident work‐hour restrictions (WHR), primarily in terms of resident well‐being, patient safety, and resident education. Most research investigating the consequences of weekly work‐hour limitations involves observational studies conducted before and after the imposition of work‐hour limits. The weekly work‐hour limit varies among countries, with the United States (U.S.) and Europe having set limits between 80 and 48 h weekly, respectively.^8^ However, there is limited literature available from Japan in this regard. Randomized controlled trials have predominantly been conducted in the U.S. and have mainly focused on the effect of shift length, rather than weekly work hours.^9^ As a result of the potential impacts and differences in healthcare systems and cultures across countries, there is currently no consistent agreement on the ideal global limits for working hours.

Given the ongoing controversy surrounding resident work hours, it is important to understand the current evidence on this topic. This comprehensive review provides an overview of the literature on resident working hours, concentrating primarily on their global regulatory landscape and the influence of their limitation, with a specific emphasis on the available Japanese literature. This review aims to help Japanese and international readers consider the optimal balance of resident working hours.

## 2 WORK‐HOUR REGULATIONS

The regulation of work hours for residents has been predominantly enforced in the U.S., Europe, and Canada, with other nations, particularly in Asia, more recently adopting similar measures (Table 1).^8^, ^10^, ^11^ However, Japan is scheduled to impose such regulations in April 2024. Most countries' regulations on resident work hours consist of two components: the total number of working hours (e.g., hours worked per week) and the longest continuous working hours in a shift. While the specific motivations for implementing WHR differ among nations and regions, they commonly prioritize medical safety, resident health, and workers' rights.^8^ This section aims to provide an up‐to‐date overview of the current status of resident WHR in Japan and other countries worldwide.

**TABLE 1:** Work‐hour regulations in Japan and worldwide.

Country	Regulation source	Maximum weekly working hours	Maximum continuous working hours	Implementation year	Reference
Japan	National laws	80 h	28 h	2024	^3^
The United States	ACGME	80 h	28 h (2017 revision)	2003	16, 19, 20
Europe	EWTD	48 h	13 h	2004 (Varies by countries)	9, 10
Canada	Local agreement by each province	60–90 h	24 h Quebec: 16 h	Varies by provinces	21, 22, 23
South Korea	National laws	80 h	36 h	2015	^24^
Taiwan	National laws	80 h (2018 revision)	32 h	2013	25, 26

Abbreviations: ACGME, accreditation council for graduate medical education; EWTD, European working time directive.

### 2.1 Japan

In 2018, the Japanese government implemented a series of measures aimed at promoting reforms in the workforce.^4^, ^5^ These reforms were primarily motivated by the decline in the working population because of falling birth rates and an aging demographic, as well as long working hours. Among the measures enacted were revisions to the Labor Standards Law, which introduced new regulations on working hours and interworking interval systems. Effective since April 2019 in large companies mainly, these new regulations have limited overtime work comprising 45 h per month and 360 h per year. In Japan, the legal working hours are 8 h per day and 40 h weekly, with any hours worked beyond these considered overtime. Prior to these regulations, there was no limit for overtime work hours in Japan.

Notably, the enforcement of the working‐hour reform for physicians (including residents) was postponed until 2024 because of the unique nature of their profession. To ensure the effectiveness of the healthcare system, the MHLW's Study Group on Working Hour Reform for Doctors has suggested setting a standard limit of 960 overtime hours per year for physicians, with the option for longer working hours in specific cases and proposing extended working hours for physicians involved in community medicine, emergency medicine, and those undergoing training.^6^, ^12^ As a result, a bill on the reform of physicians' working practices (the Medical Care Act) came into effect in May 2021.^3^ Under this bill, the upper limit for overtime work for general physicians is 960 h per year (equivalent to a 60‐h work week), which is designated as “Level A” (Table 2). Exceptions will be granted to physicians working at medical institutions providing community and emergency medical services, designated as “Level B,” and postgraduate residents and residents in specialty training seeking to acquire advanced skills, designated as “Level C.” The latter are allowed to work up to 1860 h of overtime per year (equivalent to an 80‐h workweek). To ensure health and safety, Levels B and C require no more than 28 consecutive work hours and a minimum interworking interval of 9 h.

**TABLE 2:** Work‐hour regulations among Japanese physicians.

Category	Types of physicians	Maximum annual overtime	Maximum weekly working hours	Maximum continuous working hours	Implementation year
	All workers	360 h	48 h	No limit	2019
Level A	All physicians	960 h	60 h	24 h (recommendation)	2024
Level B	Physicians in community health care	1860 h	80 h	24 h (requirement)	2024 (Until 2035)
Level B for Cooperation	Physicians dispatched to community health care	1860 h	80 h	24 h (requirement)	2024 (Until 2035)
Level C‐1	Postgraduate residents and residents in specialty training	1860 h	80 h	24 h (requirement)	2024
Level C‐2	Physicians requiring advanced clinical skills training	1860 h	80 h	24 h (requirement)	2024

*Note*: This table provides an overview of work‐hour regulations in Japan, which were obtained from a reliable source cited in reference.^3^ The table distinguishes between different types of physicians, with Levels B and C being exclusively carried out at designated hospitals. It is worth noting that Level B is scheduled to be eliminated in 2035, while the details of the reduction for Level C are yet to be determined.

It is important to note that Level C is not mandatory for all residents but is only applicable to those working in medical institutions designated by the prefecture based on actual work conditions. The proportion of residents who will take Level C has not yet been disclosed and it is feasible that only a limited number of training hospitals will be classified as Level C (otherwise, they will be categorized as Level A). Additionally, by 2035, Level B will be eliminated and integrated into Level A, whereas Level C will be gradually phased out. The details of the execution of this reduction remain undetermined.

### 2.2 The United States

In the U.S., a medical incident that occurred in New York in 1984, known as the Libby Zion case, triggered the implementation of regulations on resident work hours.^13^ This incident involved the unfortunate death of an 18‐year‐old woman as a result of a resident's medical error and brought attention to the arduous working conditions that residents face. In the aftermath, several studies were conducted and reported that prolonged working hours are associated with increased risks of medical accidents and errors.^14^, ^15^ In response to these findings, the Accreditation Council for Graduate Medical Education (ACGME) introduced a regulatory measure in 2003 to limit the working hours of residents to 80 h weekly and 30 h consecutively (24 h plus 6 h for transitions of care and education).^16^ In 2011, additional restrictions were imposed, restricting continuous duty hours to 16 h for first‐year residents, while second‐year residents and above were limited to 28 h (24 h plus 4 h). After randomized controlled trials demonstrated minimal effects on medical safety and resident burnout with relaxed continuous duty hours,^17^, ^18^ the ACGME conducted a study in 2017 to evaluate the impact of the new regulations on first‐year residents, allowing them to work for up to 28 consecutive hours (24 h plus 4 h).^19^, ^20^

### 2.3 European union

The European Working Time Directive (EWTD) has been in place since 1998, restricting working hours to ensure the health and safety of workers across all occupations in the European Union (EU). The law limits working hours to 48 h weekly, with a minimum of 11 h of rest within a 24‐h period.^8^, ^10^ Nevertheless, the degree of resident compliance with the EWTD guidelines varies in each country and has not been consistently reported. In some cases, residents can choose to opt out of the EWTD regulations, allowing them to exceed the 48‐h weekly limit.

### 2.4 Canada

There are no nationwide regulations or laws governing resident working hours in Canada.^21^ Instead, some provinces consult with provincial governments and resident unions to set guidelines for resident working hours. Currently, Manitoba, the Maritime Provinces, and Quebec have established weekly working hour limits of 89, 90, and 72 h, respectively.^21^, ^22^, ^23^ Quebec has also instituted a limit of 16 consecutive hours per week, while other provinces have established 24 consecutive hours. Each province has its own specific regulations regarding rest periods and overtime calls.

### 2.5 Asia

Concerns about extended working hours for resident physicians in South Korea led to the implementation of a law in 2015 aimed at improving their working conditions and status. The legislation mandates a maximum of 80 working hours weekly and 36 h continuously.^24^ A survey conducted by the Korean Intern Resident Association indicated a decline in weekly working hours from 114 to 88 h between 2016 and 2019, which led to increased satisfaction with training.^24^

The Ministry of Health and Welfare in Taiwan introduced guidelines for resident working conditions in 2013.^25^ The guidelines specified a limit of 88 weekly working hours, with a maximum of 32 h consecutively and a 10‐h interworking interval. Further regulations were implemented in 2018, capping weekly working hours at 80 h and mandating that residents take 1 day off per week.^26^ Moreover, since 2019, residents have been protected under the Labor Standard Act, which limits their work hours, ensures a regular day off, and provides flexibility in arranging their own working schedules.

## 3 WORK HOUR AND RELATED OUTCOMES

Residents' working hours have decreased in the past two decades, primarily in Europe and the U.S., with other countries following suit with the primary objective of safeguarding medical safety and resident well‐being^8^; however, the efficacy of such measures has yielded mixed results.^9^ Moreover, some studies have suggested that WHRs may have a negative impact on resident education and patient care. Most high‐quality intervention studies have concentrated on interventions for single‐shift lengths instead of total hours worked per week, and there is limited evidence to determine the appropriate weekly hours worked. Nonetheless, these findings offer valuable insights for Japan, which will implement WHRs in 2024. This section provides a synopsis of the consequences of working‐hour limitations, with specific emphasis on weekly hours, for resident health, performance, and education.

### 3.1 Well‐being

Extended work hours and excessive workload have been linked to adverse health effects, including mental disorders (e.g., depression and burnout), sleep disorders, cardiovascular diseases, and suicide.^7^, ^27^, ^28^ Residents' health is primarily evaluated through psychological assessments. Research indicates that residents experience higher levels of stress and burnout than attending physicians, particularly during the transition from student to resident.^29^, ^30^ While burnout is the primary outcome measured when assessing the impact of work hours and its limitations on resident well‐being, studies have also examined factors such as depression, sleep quality, and overall quality of life.

The 80‐h limit on workweeks, primarily in the U.S., is likely to have a positive effect on mental health by mitigating burnout. In 2011, a systematic review showed that five out of eight studies examining the impact of the WHR introduced in the US in 2003, resulted in decreased burnout levels, particularly emotional exhaustion.^31^ Martini et al.^32^ discovered a reduction in burnout levels from 77% to 43% among postgraduate year 1 (PGY‐1) residents after the implementation of the 2003 WHR. They also reported that, before the implementation of the WHR, those working more than 80 h weekly had higher burnout rates compared to those working less (69.2% vs. 38.5%).^33^ Similarly, studies conducted on internal medicine and surgical residents have also indicated improvements in emotional exhaustion after the implementation of 2003 WHR.^34^, ^35^, ^36^ Although these early pre‐ and post‐intervention studies were limited in size, with fewer than 200 participants, subsequent large observational studies and studies conducted in other countries have validated these findings. For instance, Ogawa et al.^37^ found that in a sample of 1241 first‐year residents in Japan, those working 80–100 h weekly, or more than 100 h weekly, had three and seven times higher risk of developing depressive symptoms, respectively, compared to those working 60 h. In a large cross‐sectional study of 6000 residents in Japan published in 2022, working more than 90 h weekly was associated with a higher prevalence of burnout and depressive symptoms.^38^ Studies conducted in Canada, France, and Taiwan have similarly reported that extended work hours (70–80 h or more weekly) for residents are linked to a greater incidence of burnout.^39^, ^40^, ^41^

Two key points warrant attention regarding the impact of weekly work hours on mental health. First, the widely implemented 80‐h‐per‐week limit may not represent the optimal threshold. Although several studies have established an association between exceeding this benchmark and compromised psychological well‐being, other investigations have revealed a dose–response relationship between work hours and depressive symptoms, as exemplified by Ogawa et al.'s study. A study published in 2022, analyzed 17000 U.S. interns from 2009 to 2020,^42^ and reported a link between longer working hours and a gradual increase in depressive symptoms, evaluated using the PHQ‐9 questionnaire. Among the interns who worked more than 90 h weekly, the increase in symptom scores was three times higher than that among those who worked 40–45 h weekly. Second, interventions on shift length may have less of an impact on residents' well‐being and mental health, as suggested by a 2015 systematic review that reported their ineffectiveness in enhancing overall well‐being.^43^ This is further supported by the results of two subsequently conducted randomized controlled trials, indicating that extending shift length did not exacerbate resident burnout.^17^, ^44^ These results were significant enough to prompt a regulatory change in the U.S., which led to the deregulation of shift length of PGY‐1 residents in 2017.

### 3.2 Performance

Inadequate sleep and fatigue from long work hours have been associated with decreased attention and cognitive performance.^45^, ^46^, ^47^ One study suggests that chronic sleep deprivation of 6 h or less is equivalent to 2 days of total sleep deprivation in terms of cognitive performance deficits.^47^ Despite expectations that limiting work hours would increase patient safety and improve medical outcomes, the available evidence is insufficient to support the conclusion that WHRs enhance medical safety, as study findings have been inconclusive.^10^, ^48^

The lack of published studies examining the association between residents' work hours and medical errors was unexpected. Landrigan et al. (2004) conducted a randomized controlled trial that compared 30‐ and 16‐h shifts for intensive care unit interns.^15^ The intervention resulted in a reduction of 15–20 work hours weekly (77–81 vs. 63 h) and a corresponding 5.6‐fold increase in medical errors among interns on the 30‐h shift schedule. Similarly, a study conducted in the UK evaluated interns working under a 48‐h‐per‐week limit and found that the intervention group working 48 h or less weekly had a significantly lower likelihood of committing errors than the control group working 56 h or less weekly.^49^ Conversely, a US‐based prospective cohort study examining the impact of work‐hour restrictions on pediatric residents found no significant changes in their sleep or work hours, or their drug prescription or self‐reported medical errors.^50^ Moreover, a cross‐sectional study investigating self‐reported practice errors among internal medicine residents found no association between an 80‐h workweek limit and suboptimal care or medical errors.^51^

Multiple systematic reviews have shown that implementing work hour restrictions for residents (2003 WHR) is associated with a decrease in patient mortality.^31^, ^52^ For instance, Fletcher et al. (2011) conducted a meta‐analysis and found that the odds ratio of patient death decreased to 0.9 (95% Confidence Interval [CI]: 0.84–0.95) after the implementation of 2003 WHRs compared to the before.^31^ However, the interpretation of these findings should be approached with caution. Baldwin et al.'s^52^ systematic review in 2011 indicated that there were no significant differences in patient mortality before and after 2003 WHRs among the teaching hospital and the nonteaching hospital groups. Thus, the reduction in mortality rates may not be a direct consequence of resident WHRs but may be influenced by other factors such as technological advancements, promotion of a culture of medical safety, and improved levels of physician practice.

Investigations have also been undertaken regarding the effect of WHRs on complications, with systematic reviews presenting inconclusive findings.^31^, ^52^, ^53^ Conversely, a systematic review conducted in 2014 on surgical residents revealed a potential escalation in complications and even increasing mortality as well.^53^ In 2017, a large cohort study documented 110 million hospitalizations before and after the enforcement of the 2003 WHRs and showed that inpatient complications surged, particularly in teaching hospitals, with a reported increase of 10%.^54^ A study comparing surgical patients before and after 2003 WHRs found a significant reduction in provider‐related complications (48% vs. 39%).^55^

### 3.3 Education

The primary objective of implementing WHRs is to enhance the working conditions of residents and improve patient safety. However, supervisors and residents have raised concerns regarding the potentially negative implications of these measures on educational aspects. The impact on education has been objectively measured through various parameters such as training opportunities, number of case experiences, and examination performance.^31^, ^53^, ^56^

A substantial body of research has scrutinized the impact of WHRs on the number of case experiences, with a specific focus on the surgical experience of surgical residents. Several studies have established that resident caseloads either decrease or remain unchanged. A systematic review conducted in 2011 examined the impact of work‐hour restrictions on caseloads and found that out of 37 studies, 11 (30%) revealed a reduction in surgical experience, while 25 showed no significant change.^56^ Out of the 11 studies that demonstrated a decrease in surgical experience, six were conducted in the U.S. and five in the U.K., implying that a less than 80‐h‐per‐week limit, whether a 48‐ or 56‐h limit, could result in a decrease in case experience. However, the sample sizes of these studies were relatively small, and the observations were short‐term. A significant study published in 2013 analyzed ACGME case logs in general surgery and reported that the number of surgical cases declined temporarily after the 2003 WHRs but subsequently recovered.^57^ Similarly, a 2014 study on thoracic surgery revealed no significant change in the number of cases before or after the work‐hour restriction.^58^ Limited data are available on whether surgical experience has recovered in the UK or Europe, but it is plausible that the 80‐h work week may have had a negligible impact on surgical experience. Additionally, a U.S. study that surveyed surgical residents' opinions following the 2003 WHRs found a decrease in case experience but an increase in reading time.^59^ This finding suggests that the quality of education might have been maintained through the acquisition of knowledge from sources other than direct medical experience.

The effect of the 80‐h work‐week restriction on exam scores has been primarily studied in the U.S., and it appears that it does not necessarily worsen scores. According to a 2011 systematic review, out of 10 studies that evaluated in‐training exam scores, two reported improvement, two reported worsening, and the remaining six found no significant differences.^31^ Moreover, a 2014 study that analyzed the U.S. Internal Medicine Board Examination showed no significant change before or after the implementation of the 2003 WHRs.^60^ A recent cross‐sectional study conducted in Japan,^61^ involving 5500 residents, revealed that resident in‐training exam scores tended to decrease when residents worked less than 60–65 h weekly and did not improve when residents worked longer. This result suggests that decreasing work hours to 60 h weekly rather than 80 may have a limited effect on exam scores.

The influence of WHRs on other educational outcomes has also been examined. Investigations on the effects of the 2003 WHRs on U.S. internal medicine residents have revealed limited engagement in educational activities.^34^, ^62^ Conversely, Jagsi et al.^63^ found that WHRs did not significantly diminish case or procedural experience, or sense of clinical preparedness among 1770 residents, despite a decrease in patient care hours from 48.5 to 42.3 h. Edson et al.^64^ reported a negligible impact on residents' learning time resulting from work‐hour restrictions. Meanwhile, a large national observational study conducted in Japan found that resident self‐study time was shorter for those working less than 60 h per week compared to those working 60**–**65 h per week, and there was no significant increase in self‐study time for those working over 65 h per week.^65^

## 4 FUTURE DIRECTIONS

**FIGURE 1:**
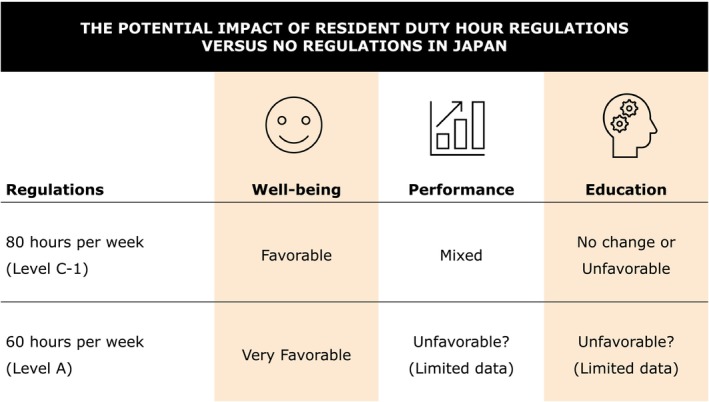
The potential impact of resident duty hour regulations versus to no regulations in Japan. Japan is poised to implement working‐hour restrictions for both physicians and residents in April 2024. The upcoming regulations will mandate different weekly working‐hour limits for residents based on their categorization as either Level C‐1 or Level A. Level C‐1 residents will be subject to a maximum weekly working‐hour threshold of 80 h in consideration of their current workload, whereas Level A residents and all physicians will be subject to a standard weekly limit of 60 h.

According to Level C‐1 regulations in Japan, the maximum weekly working hours for residents will be limited to 80 h weekly starting in April 2024. This is in line with the work‐hour limit observed in the U.S., and findings from the U.S. context may be applicable to Japan. The implementation of this regulation is anticipated to have a favorable impact on the well‐being of residents; however, it may have adverse consequences for education (Figure 1). Concerns have been raised about the educational implications, especially in relation to the reduction of case experiences, particularly in surgical settings, which has been noted in other countries. Nonetheless, the impact of such limitations may be limited in Japan as residents receive uniform training aimed at obtaining standard basic clinical skills unrelated to their specialty. The effect of restricting work hours on the occurrence of practice errors is uncertain and may not yield the desired outcomes. Positive and negative effects on mortality and morbidity have been demonstrated. However, the degree of responsibility of Japanese residents for their practice is limited, potentially rendering these effects insignificant. It is important to acknowledge that the purpose and realities of resident training vary across countries, which presents limitations when applying research from other countries to Japan. Therefore, it will be necessary to evaluate the impact of work‐hour limitations in Japan and make future improvements based on the results.

Although the exact percentage remains unclear, a considerable number of Japanese residents are expected to be subject to Level A regulations. The work week restriction of 60 h weekly is comparable to that in Europe, and there is considerable uncertainty regarding its potential impact. Research has indicated that there may be a dose–response relationship between working hours and resident well‐being and that even greater improvements in well‐being may be achieved with limits below 80 h weekly.^37^, ^42^ However, there is concern that resident education may be negatively affected, as previous research indicates that less than 60 work hours weekly may lead to decreased in‐training examination scores and less self‐study time. Therefore, it is imperative to closely monitor the situation to determine whether any adverse effects on education emerge, accounting for factors such as number of case experiences and educational opportunities. While decreased fatigue and sleep deprivation may lead to expected improvements in decreasing the incidence of medical errors, there is a possibility that the number of residents exhibiting suboptimal medical skills may increase, and this outcome warrants further investigation.

It is crucial to examine residents in specialty training (PGY‐3, 4, and 5) when evaluating the effects of resident work‐hour limitations in Japan. The introduction of a new physician specialty training system in 2018 mandated a minimum of 3 years of specialized training for physicians seeking to acquire a subspecialty.^66^ These residents may be subject to Level C regulations similar to those of medical residents (PGY‐1 and 2). However, residents in specialty training require more specialized and rigorous training and are entrusted with a significant number of decision‐making and patient care responsibilities. As such, there is a heightened concern regarding the impact of work hours on education and patient care performance. As a result of the highly individualized nature of each specialty in setting appropriate working hours, it is crucial to secure the cooperation of each specialty's associated societies.

## 5 CONCLUSION

The Japanese government announced its intention to implement weekly work‐hour restrictions for residents in 2024. The restrictions involve a combination of 80‐ and 60‐h weekly limits for each training hospital. While these measures are anticipated to enhance residents' well‐being and work‐life balance, there is apprehension that strict work‐hour limitations may compromise the quality of resident education and patient care. Despite the global acceptance of the 80‐h‐per‐week threshold following its introduction in the U.S., the incorporation of dual work‐hour restrictions in Japan is expected to represent a significant social demonstration experiment. Comprehensive studies examining the impact of work‐hour limitations in Japan could provide pivotal evidence for other countries to devise appropriate working‐hour limits.

## CONFLICT OF INTEREST STATEMENT

The authors declare that no conflicts of interest exist.

## ETHICS STATEMENT

None.
